# Supplementary material for: Characterization and management of human-wildlife conflicts in mid-hills outside protected areas of Gandaki province, Nepal
Source: PLoS One. 2021 Nov 19;16(11):e0260307. doi: 10.1371/journal.pone.0260307 (PMC8604285; doi:10.1371/journal.pone.0260307)
Supplement: Attachment — Submitted filename: Response letter.docx [file pone.0260307.s002.docx]

PONE-D-21-05727
Characterization and management of human-wildlife conflicts in mid-hills outside protected areas of Gandaki province, Nepal
PLOS ONE

Additional Editor Comments (if provided):

Q 1: Both the reviewers have raised issues related to methodology, English, readability and the use of the results. Since, the manuscript does not provide rigorous analysis and interpretation of the data. The data used in the article is insufficient for making such conclusions. It is irrelevant to clubbing of a snake bite with large carnivores which makes the findings little more complicated. Moreover, the authors failed to provide methods clearly in methodology section which is making me difficult what analysis and how they have used the analysis technique. Hence, I suggest to resubmit the article after addressing the comments of both the reviewers as a new submission.


*A: Response: Thank you editor, Dr. Sharma. We have gone through the MS, checked the errors and edited as required and suggested. Please find below the detail corrections.*

Review Comments to the Author
Please use the space provided to explain your answers to the questions above. You may also include additional comments for the author, including concerns about dual publication, research ethics, or publication ethics. (Please upload your review as an attachment if it exceeds 20,000 characters)

REVIEWER #1: The authors have highlighted an important issue of man- wildlife conflicts, however, the manuscript needs further improvement in the form of presenting the facts and clearing about the methodology.

Q 1: The species considered in the data set are from different genera, the clubbing of a snake bite (which has been referred as injury in the submitted manuscript) to the conflict incidences of Common leopard and Asiatic black bear is not advisable. either the authors present the data of each species individually, so that interpretation of collected data is presented in a meaningful manner.

*A: Thank you editor and reviewers for this nice comment. Now we also came to know that clubbing snake was erroneous and we separated and discussed the results independently. Snake was mentioned only in lines 224 and 279.*

Q 2: The classification on the basis of caste may not be advisable, and in the discussion part, there has been no considerable impact found based on the results.

*A: We appreciate the concern but the HWC in mid-hills of Nepal is season and site specific and largely based on ethnicity. Most of the ethnic groups and dalits (scheduled, untouchables) live at the forest fringes and even some of the ethnic groups are swidden farmers adopt the slash-burn practices, often become victim of HWC. Despite statistically insignificance, the account based on ethnicity in our result has difference and it is worth discussing. Most of the local readers expect the HWC and ethnicity to be discussed.*

*Line 246-247*

Q 3: The references to back the results may be from the same species and if possible from the similar landscapes e.g, black bear behaviour and ecology are different than that of the sloth bear or American black bear, so comparing the results with other species may not be helpful.

*A: Thank you for suggestion in making the article more pertinent. Now we removed the irrelevant literature and used local and that of neighboring region.*

*Now we used references (Charoo et al 2011; NTNC 2013, Sharma et al 2015;) that account the fate of black bear. Line 394-404, 458-460*

Q 4: The statistical analysis needs a revision, the Chi-square tests and Probability index results need to be interpreted accordingly.

*A: We reviewed the statistical tests/methodology and conducted tests and analyses following references and experts idea. Line 188-199.*

REVIEWER #2: Review PONE-D-21-05727

Q 1: The article presents pattern of Human-wildlife conflicts in Nepal, based on records from forest department and certain level of ground truthing. This is important information, which can help management of wildlife in Nepal and neighboring countries. However there are certain improvement areas that needs to be addressed before publication. Major issues are:
1: Redundancy and repetition (particularly in discussion section) and language issues. Discussion is not clearly linked to results, it needs to be summarized to present the main message.

Redundancies are removed and sentences are edited.

*A: Discussion are made direct to the results heading.*

Q 2: Authors have considered several factors in evaluating conflicts on temporal and spatial scales. However in analysis they have relied on univariate analysis and tested effect of each variable separately, and shifted from one test to other for different variables. This is not an effective way, and results could be misleading. For example, they have used GLM for testing Distance from forest edge, and Chi-square test for many variables in Table 2. I suggest all variables should be tested in single analytical framework, eg GLM.

*A: We tried GLM for all variables but none of the variable showed the result. Then we segregated the variables into Spatial, temporal and socio-demographic and the former was assessed with GLM and the latter two with Chi-Square. Line 188-194, 196-199.*

Q 3: Article mostly discusses circumstances of wildlife attacks, it would be useful to discuss fate of animals involved in attacks and evaluate management action of the forest department. Since authors have interviewed affected families, one would have expected documentation of their reactions towards attacks and losses they suffered.

*A: Thank you for raising an important issue. The issues were discussed line 458-470.*

Q 4: Many acronyms like DFO, NTNC, are used without defining them

*A: Now defined them. Line 155, 312*

Q 5: Formatting issues need to be addressed

*A:Without line numbers, it is hard to make specific comments, but some are below:*

*Line number added.*

Q 6: Page 6, replace “reported by leopards’ with attacked by leopards

*A: Replaced as suggested, line 259.*

Q 7: Page 6, “Tanahun district was found the most susceptible”, replace susceptible with vulnerable

*A: Replaced as suggested Line 263.*

Q 8: Tabl 1, abbreviations should be defined

*A: We replaced that table by a new one. It does have abbreviations with their definitions.*

Q 9: Page 9, Effect of outmigration on conflict is not clear

*A: Now the result of outmigration is discussed. Line 345-348*

Q 10: Page 9, statement “Most of the attack sites (human settlement area and agriculture lands) were found within the one km distance from the nearest forest, despite the distance between forest” contradicts GLM results. Why

*A: This statement was corrected and discussed appropriately. Line 357-361*

Q11: Page 9, paragraph 3 needs rephrasing

*A: The content was rephrased according to the changed analyses. Line 375-379.*
