# Supplementary material for: Characterization and management of human-wildlife conflicts in mid-hills outside protected areas of Gandaki province, Nepal
Source: PLoS One. 2021 Nov 19;16(11):e0260307. doi: 10.1371/journal.pone.0260307 (PMC8604285; doi:10.1371/journal.pone.0260307)
Supplement: Attachment — Submitted filename: Final MS.2..docx [file pone.0260307.s003.docx]

**Characterization and management of human-wildlife conflicts in mid-hills outside protected areas of Gandaki province, Nepal**

Kedar Baral^1,2^, Hari Sharma^3^, Bhagawat Rimal^4^, Khum Thapa-Magar^5^, Rameshwar Bhattarai^6^, Ripu Kunwar^7,*^, Achyut Aryal^1^ and Weihong Ji^1^

^1^Institute of Natural and Mathematical Sciences, Massey University, New Zealand

^2^Division Forest Office, Kaski, Department of Forest and Soil Conservation, Nepal

^3^Central Department of Zoology, Tribhuvan University, Nepal

^4^College of Applied Science (CAS-Nepal), Tribhuvan University, Nepal

^5^Colorado State University, Fort Collins, CO, USA

^6^The Asian Center for Development, Kathmandu, Nepal

^7^Florida Atlantic University, Boca Raton, FL, USA

^*^Author for correspondence (email: rkunwar@fau.edu)

**Characterization and management of human-wildlife conflicts in mid-hills outside protected areas of Gandaki province, Nepal**

**Abstract:**

Understanding the human wildlife conflict (HWC) is important because it can help mitigate such conflicts and promote wildlife conservation. In this study we investigated the spatial distribution of human casualty caused by wildlife, temporal and seasonal trends of wildlife attacks and perception of local people towards wildlife conservation in the mid-hills outside protected areas of Gandaki province, Nepal. Data were obtained from HWC records from the forest offices of five divisions, Parbat, Lamjung, Syangja, Tanahun and Kaski (PLSTK), and interviews of local people between March and December 2019. Supplementary data were collected from the secondary sources. We have recorded 77 cases of wildlife attacks (69 human injuries and 8 mortalities) during the period of nine year between 2011 and 2019. The number of wildlife attacks increased over this period, and 50 % attacks occurred in winter season (χ2 = 13.39, df = 3, *p* < 0.05). Leopard (*Panthera pardus*), and Himalayan black bear (*Ursus thibetanus*) were the major species involved in these conflicts. Leopard was the most feared species that causes highest number of human mortalities; the most severe type of HWC outcome. Most attacks occurred when people were working outside of their house or ventured into forests for extraction of forest products. Through this study, we were able to provide a better understanding of spatial, temporal and causative patterns of wildlife attacks, impact, and some mitigation measures. Such knowledge is important for decision making in HWC mitigations and better human-wildlife co-existence.

**Keywords**: Leopard, Human injury, Conflict, Conservation, Nepal

**Introduction**

The Human-Wildlife Conflict (HWC) refers to the interactions between human and wild animals that results in negative consequences on livelihood and life of people and or wild animals [1]. HWC dates back to human prehistory. The earliest forms of HWC occurred in the form of predation of early hominoids by wild animals [2]. HWC occurs in different contexts involving a range of animal taxonomic groups [3] [4] [5]. It is a common issue in the Himalaya region where wildlife and people co-exist [6] and share the limited resources [5]. This region exhibit great propensity for HWC due to its rich biodiversity, heavy reliance of people on forests, cropland and animal husbandry for livelihoods [7] [8]. A high degree of dependency on forest ecosystems and prevalent poverty has led to unsustainable extraction of forest products and conversion of forests into agricultural land [9]. HWC has been complicated as poaching, deforestation, habitat degradation and fragmentation and overexploitation are escalated [10] with change in human population [11]. With increasing and inevitable threats posed by the changes and unsustainable exploitations, both human and wild animals as well as livestock combined with wildlife habitats are threatened and the HWCs are outstripped by folds [12].

The most common forms of HWC in the Nepal Himalaya are crop raiding, property damage, livestock depredation and wildlife attacking human (thereafter shortened to “human attacks”) and causing injuries and mortalities [12][13][14]. Among these types of HWCs, the latter two have the highest cost [15] [16]. Leopard (*Panthera pardus*) causes the highest number of human attacks in Nepal, followed by wild elephants [6]. The occurrence of leopard attacking humans in Nepal is higher than that of anywhere else within leopard distribution [17]. The livelihood of rural people has been severely impacted by increasing attacks and predation by wildlife [18] and ruining traditional livelihood practices [19][20].

Most of the protected areas (PAs) in Nepal are established to conserve large mammals. After establishment of PAs, the HWC caused by the large mammals such as elephants, rhinos and tigers has been increasing [21]. The increased competition among animals within PAs may have triggered the invasion of wildlife to areas outside PAs, resulting in frequent HWCs [7]. In the last five years, over two third of HWC incidences in Nepal occurred outside of PAs [22][23]. Due to the successful implementation of community-based forestry programme in the last three decades, degraded forests have been restored in mid-hills outside PAs, promoting forest cover and free movement of wildlife [24] [25]. Such situation has increased the encounter of local people with wildlife and HWC increased as a result. Leopard attacks occurred almost entirely outside the protected area system (PAS) and mid-hills of Nepal [26][27]. Moreover, the landscape outside PAs are human dominated and the HWC is mostly associated with the local livelihood and cultural values [28]. HWCs occur in human settlement outside PAs while people carrying out activities include farming, livestock herding, and harvesting forest products [2][13][29]. Summer grazing, herding and collection of plants from forests and fringes are still persistent among the Gurung ethnic people of Parbat, Syangja and Kaski districts [30]. Gurung ethnic people are mountain dwellers, herder of sheep, swidden farmers and trans-himalayan traders [31] often confront with wildlife in the mountain rangelands. The management of HWC at landscape level addressing the issues outside and inside PA are limited [32]. Maintaining suitable habitats and populations of wildlife outside PAs and increasing independency of livelihood on forests are therefore urged [33].

The increasing severity and complexity of HWC is thought to have been caused by a number of factors, such as expansion of human activities into wildlife habitats, recovery and expansion of natural habitats and wildlife populations [34][35][36]. Even though the conflict jeopardized the coexistence of human and wildlife, the studies on HWC analyzing the causes, pattern and characteristics and those from mid-hills and central Nepal and outside PAs are scarce [15][23]. At outside PAs of Nepal, the Himalayan black bear (*Ursus thibetanus*) and leopard (*Panthera pardus*) are the major mammals responsible for human attacks [37][38]. However, the ecology and human interactions with these animals are poorly studied. Understanding the patterns and causes of HWC in the areas outside PAs of Nepal is highly pressing and most pertinent in formulating conservation policies to mitigate HWC and improving the local livelihood. In this connection, we examined the extent and magnitude of conflict between wild animals and agro-pastoralists reported from outside the PAs of Nepal. In this study, the conflicts associated with wildlife and human attacks were evaluated and we aimed at (1) assessing the spatio-temporal distribution of HWC leading to human casualties, and (2) recommending the measures for future conservation planning.

**Methods**

**Study area and site description**

Five districts, Parbat, Lamjung, Syangja, Tanahun and Kaski (hereafter referred as PLSTK) (27˚55’ - 28˚26’ N, 83˚58’ – 84˚25’ E) were selected as study area (Figure 1). These districts have human population of about 1.4 million, many of whom live in remote and isolated areas with poor access to markets and easy access to forest resources. These five districts cover the area of approximately 7,000 km^2^ and represent lowlands (60 to 1000 m asl), mid-hills (1000 to 3000 m asl) and high mountainous area (3000 m to 5000 m asl) of central Nepal. Mid-hills have temperate type of bioclimatic features. The high mountainous part of Kaski and Lamjung districts shares 11% of Annapurna Conservation Area (7,629 km^2^), the largest PA of Nepal.

Within the study sites, Devghat, Tanahun district represents as the lowest elevation and tropical bio climate (187 m asl) whereas the Lwang-Ghalel (~ 2500 m) of Kaski district, Chimkeswari (2325 m) of Tanahun, Panchmool (~ 2600 m) of Syangja district, Dahare-Deurali (~ 2600 m) of Parbat district and Bahundanda (~ 2600 m) of Lamjung district represent the highest elevations and temperate climates. The study sites cover a big section of Chitwan Annapurna Landscape (CHAL) corridor. The CHAL represents north-south corridor and connects alpine high mountains in north to tropical lowlands in south through Gandaki watershed. The Gandaki watershed connects Marsyangdi (Lamjung), Madi (Tanahun), Setigandaki (Kaski) and Kaligandaki (Parbat and Syangja) sub-river basins. These sub-river basins boasts high biodiversity and supports rich natural, social and cultural heritage. It is an important transit route for migratory birds and is home to endangered species like leopard, red panda (*Ailurus fulgens*) and Himalayan black bear [36][39].

**Figure 1: Location of study area showing the districts, elevation and points of wildlife attacks. Data for this map was downloaded from ICIMOD [40]. https://doi.org/10.1371/journal.pone.0246390.g001**

**Data collection**

We received human ethics research approval 4000023041 from Massey University New Zealand for our research (Supplementary file 1). Prior to fieldwork and interview, we also received written consents from the division forest offices and oral consents from participating individuals. HWC data of the study districts were collected from both published articles and grey reports. HWC databases of PLSTK division forest offices between 2011 and 2019 were reviewed. Data records only after 2010 were logged when the official recording of wildlife damage for compensation scheme was formally started. A total of 77 cases of human attacks (injury and death) were recorded based on the accounts of PLSTK division forest offices (DFO). Divisional forest officers were consulted to crosscheck the data and gather supplementary information. Field visit was made to meet the victims and persons accompanying the victim, and to observe the sites of attack. We visited the sites of 12 attacks including all eight human deaths. Since Tanahun district has high incidents of human-wildlife conflict, we visited all six sites (Nareshowar tar, Purkot, Khahare, Bhansar, Samjur and Mirlung) of human death in Tanahun. Field observations and checklist surveys were carried out between March and December 2019 to verify the sites and conflicts, and to collect additional information regarding the perception of people and management options. We took 36 interviews with the victims and another 21 interviews with the persons accompanying the victim during the attack, or an immediate family member well aware of attack details. Confidentiality of each case was maintained during the interview and analysis process by recording a code in each case and keeping the interviewees anonymous. We used a semi-structured checklist to conduct the interviews. Interviews were conducted in Nepali language with the help of a forest ranger and local assistants.

**Data analysis**

Variables associated with human-wildlife attacks of the districts were mainly four types; temporal, spatial, socio-economic and wildlife species involved (Supplementary file 2). Temporal variables include year, month, season and time of attacks. The attack time was classified into six periods (0-4, 5-8, 9-12, 13-16, 17-20 and 21-24 hours). Similarly, spatial variables were district, elevation, distance between point of attack and forest, and land use (forest, agricultural land, road and settlement). The distance from the casualties and nearest forest edge was measured in Google Earth in order to test whether the HWC is a function of distance. Socio-demographic variables were the ethnicity, gender, and age group of the victims. The data associated with demography and socio-economy was aggregated and decoded for confidentiality of the respondents.

For statistical analyses, qualitative data were recorded as ‘1’ for yes and ‘0’ for no, and a comparison between data sets regarding the number of the events and with different variables were made in terms of percentage. We conducted multivariate logistic regression with the entire independent variables [total of 11 variables: temporal (4), socioeconomic (3), spatial (4)] in the model to understand the relationship between predictive and explanatory variable (human death and injury due to wildlife attack). However, no single variable independently predicted the attacks in the analysis, and this could be a reason of the large volume of dataset, and the diverse nature of variables.

For convenience, we conducted two different statistical approaches following Naha et al. [41]. First, we conducted chi-square test of independence to understand the association between temporal (year, month, season and time), and socio-demographic variables (ethnicity, gender and age) with the wildlife attacks. We classified victims into four age groups (< 20, 21-40, 41-60 and > 61 years), and three social groups (Brahmin-Chhetri, Ethnic and Dalits) based on their caste/ethnicity. The association between socio-demographic and temporal variables and the attacks were analyzed using Pearson chi-square test. For the spatial dataset, we conducted a generalized linear model with binomial distribution to predict the effect of variables on the wildlife attacks following Acharya et al. [6]. We used a priori candidate model and ranked them based on Akaike Information Criterion (AIC) values. Those models with lowest AIC values were considered the appropriate for explaining the wildlife attacks. A model with a ∆AIC (the difference between the two AIC values being compared) of ≤ 2 is considered significantly better than the model it is being compared to. The data were summarized in terms of mean (M) and percentage, and the measures of variability recorded in terms of SE and at confidence interval of 95%. Statistical analyses were undertaken in R statistical software (R Core Team 2016; Version 1.0.44).

**Results**

***Human wildlife conflict***

Seven types of conflicts (crop raiding, livestock depredation, human attacks, traffic collision, disease transmission, property damage and mental distress) and associated 663 cases were recorded in studied districts between 2011 and 2019, based on our review and field observations. Among these HWC cases, livestock depredation and crop raiding were major prevailing events. There were 77 (12%) cases pertaining to attacking human by wildlife, and were used for further analyses (Figure 2). Out of the 77 cases, 67 (87%) were involved with Leopard, nine with Himalayan black bear and one with a snake *(Naja brasiliensis* [*laurenti*](https://en.wikipedia.org/wiki/Josephus_Nicolaus_Laurenti)*)*.

**Figure 2: Types of HWC resulting in human casualties and injuries**

***Temporal variations of attacks***

The mean number of humans attacks per year was 8.55±1.15 (injuries 7.66±1.13 and the deaths 0.88 ± 0.84). Distribution of human attacks was increasing over the period of nine years, (χ2 = 0.232, df = 76, *p* > 0.05) (Figure 3). The frequency of human attacks varied with month and season (χ2 = 152.08, df = 76, *p* < 0.001, and χ2 = 13.39, df = 3, *p <* 0.05 respectively) with highest frequency of human attacks occurred in winter (December-March) and lowest in summer (July-August) (Figure 3). There were 42% attacks between September and December. The frequency of attack varied with time of the day (χ*2* = 134.45, df = 76, *p* < 0.001). The highest percentage, 52% attacks, was recorded between 15.00 pm and 19.59 pm.

**Figure 3: Temporal distribution of HWCs in study area**

***Socio-demographic characteristics of victims***

Of the 77 victims, 79% (n = 61) were men and 21% (n = 16) were women. There was no difference in the proportion of male and female victims (χ2 = 0.59, df = 1, *p* > 0.05). There were no significant differences in the distribution of attacks among the ethnic groups (χ2= 4.52, df = 2, *p >* 0.05). This shows that the attacks by wild animals in PLSTK districts have been noted to occur independent to gender and ethnicity. Victims’ ages ranged from 3 to 84 years at the time of attack. Most victims (42%, n = 33) were 41 to 60 years old followed by 24% (n = 19) 21 to 40 years old. The chi-square test showed a significant difference in the age groups of the victims (χ2 = 24.11, df = 3, *p* < 0.05) (Table 1).

Table 1. Association between socio-demographic, ecological variables and human wildlife conflict. df = degree of freedom.

| **Variables** | | **Attacks (N = 77)** | | **Coefficients** | | |
| --- | --- | --- | --- | --- | --- | --- |
|  |  | **Death (n)** | **Injured (n)** | **Chi-square** | **df** | ***p* value** |
| Gender | Female | 3 | 13 | 0.594 | 1 | *0.440* |
|  | Male | 5 | 56 |  |  |  |
| Ethnicity | Brahmin/Chhetri (B/C) | 2 | 41 | 4.521 | 2 | *0.104* |
|  | Dalit | 3 | 9 |  |  |  |
|  | Ethnic | 3 | 19 |  |  |  |
| Age | 0-20 yrs | 6 | 6 | 24.118 | 3 | *0.002* |
|  | 21-40 yrs | 0 | 19 |  |  |  |
|  | 41-60 yrs | 1 | 32 |  |  |  |
|  | 61-84 yrs | 1 | 12 |  |  |  |

***Human attacks at spatial scale***

Between 2011 and 2019, eight people were killed and 69 were injured in five districts. All eight human deaths were attacked by leopard in Tanahun (n = 6) and Syangja (n = 2). Of the 77 human casualty cases, 6 human deaths and 16 injuries were reported from Tanahun followed by 18 injuries in Lamjung and 16 injuries and 2 deaths in Syangja. While the lowest record (n = 5) was reported in Parbat district. Inside district, Bhanu municipality of Tanahun district was found the most vulnerable and six children were killed by leopards within a period of two years between 2018 and 2019. Besides Leopard attack, Tanahun and Lamjung districts were also vulnerable to bear attacks (Table 2) however, these attacks were mostly inside forest. In contrary, the leopard attacks were recorded mostly from nearby of settlement and agriculture lands. Total 48% (n = 37) attacks were reported at human settlement areas. There were 27% attacks in agriculture land (n = 21) and 24% (n = 19) in forest. The probability of human attacks was most likely to occur in decreasing elevation and distance from the forest (Table 2).

Table 2. Akaike Information Criterion (AIC) scores of generalized linear models with binomial structure predicting human attacks by wildlife in Parbat, Lamjung, Syangja, Tanahun and Kaski (PLSTK) districts, Nepal. df = degree of freedom, ∆=delta.

| **Models** | **Df** | **AICc** | ∆**AIC** |
| --- | --- | --- | --- |
| District | 5 | 49.2 | 0 |
| District + Distance from forest | 6 | 50.9 | 1.71 |
| District + Elevation | 6 | 51.2 | 2.02 |
| District + Land use | 8 | 51.4 | 2.21 |
| Elevation | 2 | 51.5 | 2.36 |
| Elevation + Land use | 5 | 52 | 2.83 |
| District + Elevation + Land use | 9 | 53.1 | 3.93 |
| District + Distance from forest + Elevation | 7 | 53.2 | 3.97 |
| Null | 1 | 53.4 | 4.24 |

**Discussion**

***Human attacks***

We reported only three wild animals (Leopard, Himalayan black bear and snake) from five districts (Parbat, Lamjung, Syangja, Tanahun and Kaski) attacking humans while there are 26 wildlife species causing human-wildlife conflicts and 13 triggering severe human attacks in Nepal [42][43][44]. Of the animals, leopard attacked 67 and Himalayan black bear injured nine humans hinted that the leopard attacks were not accidental and severely threatened local people and their livelihood. The studies in different parts of the mountain areas of the world found that Himalayan black bear and leopard are the major mammals responsible for human attacks [37][38][44][45][46][47]. The big mammals are potentially dangerous in causing property destruction and inflicting injuries to people else where [48]. There were 441 leopard human attacks in Nepal over a decade between 1994 and 2004 [47]. Over two third leopard attacks were recorded from human-settlement areas and agriculture land.

A total of 77 human attacks including 89% injuries and 11% deaths were recorded in five study districts of central Nepal over a period of nine years between 2011 and 2019. The average number of attacks per year was 8.55 (SE 1.15), including 7.66 (SE 1.13) injuries and 0.88 (SE 0.84) deaths. Similar finding of average seven injuries in a year was recorded in Chitwan district, central Nepal [21]. The records in our study area were less than that of Pauri Garhwal, India where the average injuries and death per year were 11 (SE 1.13) and 3 (SE 0.6) respectively [49]. The higher result in Pauri Garhwal is probably due to the higher population density (130/sq km) than our study area (110/sq km). The Pauri district (area 5230 sq km) of India joins with the western boarder of Nepal having similar mid-hill types of physiography and subtropical types of climate. Similarly, the forest cover in Pauri is higher (64%) than our study area (32.5%), which may promote the population of leopard.

Human attacks by Himalayan black bear are considered to be the most ubiquitous form of conflict in the mid-hills of Nepal [44]. All nine attacks by Himalayan black bear were recorded at Tanahun and Lamjung districts. Similarly, within the Panchase Protected Forest, which lies across three study districts (Kaski, Syangja and Parbat) four cases of human casualty were associated with Black bear [23]. Panchase is the area where the agro-pastoralism is still prevalent. Human casualty is common in mid-hills where the livelihood is agro-pastoralism. A report of Nepal Trust for Nature Conservation (NTNC) of a study from 2005 to 2013 [46] catalogued six cases of Himalayan black bear attacks in Manaslu Conservation Area, Gorkha, Central Nepal.

***Distribution of HWC***

Our study area is among the areas of highly vulnerable to HWC. Despite the vulnerability, the human attacks in our five study districts were varied with magnitude. There were eight death reports, all from the leopard attacks and only occurred in two districts (six in Tanahun and two in Syangja). In Nepal, 75 out of 77 districts have been suffering from HWC, with Jhapa, Baitadi, Tanahun, Kaski, Arghakhanchi, Manang, Mustang districts being the most suffered [47]. Of the 77 human casualty cases, 22 were from Tanahun followed by 18 from Lamjung and Syangja each, 14 from Kaski and the lowest record (n = 5) was from Parbat district. The probability of human attacks was most likely to occur in Tanahun district since the district is characterized with low elevation range contained with high forest and habitat degradation and livestock holding size. Tanahun district has the highest livestock holding size (3.72) compared to the rest districts [50]. There were 12 people killed by leopard in Kaski district between 1987 and 1989 [51] uncovered that the HWC is prevalent and persistent in mid-hills. Tanahun and Syangja districts truly represent the mid-hill physiography are found to be the most susceptible for HWC. Leopard is commonly inhabited mid-hills and the snow leopard at mountains and the Himalayas [52]. Thapa [53] reported that 45 humans were attacked and 14 were killed by leopard in Chitwan Annapurna Landscape (CHAL) in a period between 2006 and 2013. There were seven human injuries made by leopard in 2009 in Chitwan district, central Nepal [21]. There were 20 confrontations of leopard in Kathmandu valley in a period of three years between 2010 and 2013 [54].

All these incidences revealed that the central Nepal and mid-hill districts are highly vulnerable to leopard attack and the patterns of wildlife attacks on humans are associated with spatial factors. The forest habitat of the mid-hills is surrounded by the large human population. Over 50 percent population of the country lives in the mid-hills and it is complicated by persistent outmigration to the cities. The hills and mountains not only have the highest percentage of poverty (42.3%) and outmigration on average, but they are also increasing [55]. The local poverty and outmigration aggravate HWC at local scale as rural residents have a greater appreciation and more utilitarian attitude towards wildlife [56]. Outmigration caused the lack of working human resource in farmland resulting in land abandonment. Fallow and abandoned land promotes the growth of ruderal shrubs and bushes [57]. Wildlife lurks in the bushes and shrubs in search of food since the wild prey species are declining over time.

Half of the wildlife attacks (48%) we reported were from human settlement areas followed by that from agriculture land 27% (n = 21) and forest 24% (n = 19), revealing that the most cases happened in anthropogenic landscapes. Generally, the risk probabilities by large carnivores are reported to increase with dense forest [58] but our results suggest that risk of human attacks by wildlife are towards human settlements. There were 45% leopard attacks in settlement areas followed by 22% in agriculture land. Most of the leopard attack sites (human settlement area and agriculture lands) were found within one km distance from the nearest forest. This may be because that leopard is adapted to live in the forest fringes, nearby human habitations and moderate vegetation cover [49][59] and avoid confronting with bigger carnivore mammals such as tigers [60]. Leopard tolerate proximity to humans better than bears, lions and tigers and often come into conflict with humans [61]. Greatest number of attacks in Tanahun district (n = 22) could also be attributed to the livestock size and forest degradation [62].

Due to the presence of settlement areas of poor people especially in small hamlets, inside and adjoining to the forest area has been increasing the probability of encounter of people and wildlife. On the other hand, due to the wider spread poverty in such area people are collecting forest-based fruits, vegetables, nuts etc. and poaching the wildlife to sustain their livelihood. Studies show that poaching of prey species and habitat degradations are rampant resulting in different HWCs in Nepal [6][26][53]. Moreover, habitat degradation and less awareness about wildlife conservation in the areas also led the conflict. There is likely to have increasing HWC in mid-hills due to higher dependency of people on forest and the area is extensively extended outside protected area system. Thus the spatial features are more useful in prediction of HWC in PLSTK districts.

***Temporal Pattern of HWC***

The frequency of human attacks varied with time, month, season and year. There were 52% attacks between 15.00 pm and 19.59 pm in study area conforms with the pattern of leopard attack in Pauri Garhwal where the most attacks (53%) were reported during day time (08.00 am –16.00 pm) [41]. The leopard also attacks early and late night [64]. Thus leopard remains active in down and dusk. We recorded 31% leopard attacks (n = 24) between 17.00 pm and 19.59 pm supporting the crepuscular nature of leopard. About 56% human attacks were happened between autumn and winter season. This finding may be associated with the patterns of frequent movement of human for crop harvesting and forest product collection. The increased human activities was reported as a primary reason of rising leopard attack in India [63].

Most of the attacks made by Himalayan black bear occurred between October and February. Our result substantiated the earlier results [29][65] which reported that the winter is likely to have greater number of attacks by Himalayan black bear in the mid-hills. This coincides with the range shift of black bear from snow covered high elevation to mid-hills. Frequency of bear attacks is also associated with people’s visitations to forest. High number of attacks recorded in our study area coincided with higher visitation of people to forest for collect forest products and firewood. Attacks by Black bears during late August to September were recorded high in the Sichuan Province in China coincide with wild mushroom harvesting [66]. The majority of the crop depredations by black bear in India (70%) occurred between August and September [67]**.**

***Characteristics of victims***

Out of eight human killings by leopard, six people were aged below eight years. We found that the average age of the victims attacked by wildlife in and around settlement was 37.6 years and in forest area was 43.8 years. The middle-aged people (41-60 years old) were attacked more because they were more likely to engage in outdoor occupations. Generally male are engaged in outdoors and they were found to be frequently attacked (61, compared to 16 of females) by wild animals. However, the wildlife attack was indifference to gender and ethnicity. Farmers were the major victims of animal attack. The farmers involved in household activity were frequently attacked followed by individuals involved in forest product collections. Those who frequently exploit forest for their livelihood and pass by the forest for commuting were frequently been attacked (39% of total cases) by wild animals.

***Causes and impacts***

Human attacks were found increasing in recent years. There is an increasing trend in the number of human attacks due to wildlife interactions, even in the areas with no previously reported incidents [13][14]. This trend was consistent to increasing forest cover and PAS management [23]. We reported an increasing forest cover (388 ha/yr) in the PLSTK districts between 1996 and 2016. Increasing forest cover was also accounted between 2000 and 2010 in CHAL districts [68] and similar accounts of increment was reported by Baral et al. [69]. There were several reports of increasing HWC as increasing forest cover [70], attributed by the successful community-based forest conservation programs in the mid-hills and free movement of wild animals to nearby agricultural lands [25][71][72][73]. Successful community-based forestry programs led increased forest cover and wildlife movement.

In the mid-hills of Nepal, animal husbandry, forest product collection and agro-pastoralism create competition between local communities and wildlife for the use of natural resources. The situation was worsened as the land-use change resulted population decline of prey species, resulting in increased incidence of wild animal preying on livestock and escalated HWC. Forest is a major source of rural livelihood. Firewood and fodder make up nearly 75% of the energy supply and 37% of the livestock feed, respectively and these are mostly harvested from forests [74]. Collection of forest products for local livelihood and primary healthcare requires the person to venture several kilometers into the forests; thereby the chance of encounter with wild animals is very likely. Ruining traditional harmonious relations between wildlife and local people prop frequent HWC [75].

Nowadays, because of the increasing urbanization and land use change [35], land abandonment and unattendance of agricultural lands transformed areas into shrubland covered by invasive species [76]. These invaded areas enabled wild animals to approach and lurk closer to human settlement and result the HWC. The majority of HWC occurred in nearby human-dominated landscapes highlights the need for proper management of areas outside PAS.

***Human-Wildlife conflict management***

There are some mitigation strategies attempt to reduce the level of impact and improve tolerance and sanctions imposed for retaliatory behaviors [77]. The most efficient ones are incentive programs, compensation systems, habitat management and community-based natural resource management schemes [2]. Despite the compensation system against wildlife attacks started for eight years, the procedures of compensation are arduous and the compensating the human values in money is unappeasable. Without taking into account of spatio-temporal variability and fate of wildlife seem to be ineffective in mitigating conflicts on a long-term basis [45] [63]. Thus, a simple procedure of compensation, and reduction of human dependency on forests in sustainable manner by providing alternate livelihood options can help reduce HWC and improve the livelihood of rural communities. Habitat management combined with HWC mitigation would achieve better conservation outcomes. This requires creating strategies, integration and implementation of government programs and active participation of local communities. In order to develop relevant mitigation strategies, adopting traditional knowledge and participatory approaches that acknowledges the ideas of local people affected by wildlife deemed necessary. Land-use planning and well planned resettlement of people to another sites from highly HWC vulnerable area and inclusion of mid-hill forests in PAS might be helpful in wildlife conservation and mitigation of HWC in long run.

**Conclusions**

Based on the findings of present study, we conclude that patterns of wildlife attacks on humans are influenced more by spatial factors. Leopard was the major wildlife cause HWC followed by black bear. The conflict ranged from crop raiding, livestock depredation, traffic collision, property damage, transmission of diseases and human attacks. Among them, human attack was the most critical expression of HWC and needs addressing sensitively to liaison the local support for wildlife conservation.

Distribution of human attacks was not significantly different from expected over the period of nine years, however, the distribution of attacks was found increasing. The majority of attacks were occurred in and around human-dominated landscape, followed by forest. People lamented that the HWC in these districts is chronic and hinted that the decline in wild prey fueled by land use change is regarded as a primary driver of human attacks. Thus there is an urgent need for proper management HWC where the large number of human population inhabiting near forests is highly vulnerable to wildlife attacks. Understanding the causes of human casualties and reducing their incidence are imperative. Removing bushes and invasive species around human settlements to be precise should be initiated to restore forests and reduce wildlife attacks. The community-based forest conservation to yield sustainable supply of forest products and improve prey population is crucial in HWC management.

**Acknowledgement**

We thank study area residents and DFOs who responded to our queries and survey. S Bhandari, B Adhikari, and 2 anonymous reviewers provided valuable comments on earlier drafts of this manuscript.

**References**

1.	Attia TSN, Martin T. N, Forbuzie TP, Angwafo TE, Chuo MD. Human Wildlife Conflict: Causes, Consequences and Management Strategies in Mount Cameroon National Park South West Region, Cameroon. Int J For Anim Fish Res. 2018;2: 34–49. doi:10.22161/ijfaf.2.2.1

2.	Ogada MO, Woodroffe R, Oguge NO, Frank LG. Limiting Depredation by African Carnivores: the Role of Livestock Husbandry; Limitando la Depredación de Carnívoros Africanos: el Papel de la Crianza de Ganado. Conserv Biol. 2003;17: 1521–1530.

3.	Baruch-Mordo S, Breck SW, Wilson KR, Theobald DM. Spatiotemporal Distribution of Black Bear–Human Conflicts in Colorado, USA. J Wildl Manage. 2008;72: 1853–1862. doi:10.2193/2007-442

4.	Okello MM. Land use changes and human–wildlife conflicts in the amboseli area, kenya. Hum Dimens Wildl. 2005;10: 19–28. doi:10.1080/10871200590904851

5.	Carter NH, Shrestha BK, Karki JB, Pradhan NMB, Liu J. Coexistence between wildlife and humans at fine spatial scales. Proc Natl Acad Sci U S A. 2012;109: 15360–15365. doi:10.1073/pnas.1210490109

6.	Acharya KP, Paudel PK, Neupane PR, Köhl M. Human-wildlife conflicts in Nepal: Patterns of human fatalities and injuries caused by large mammals. PLoS One. 2016;11: 1–18. doi:10.1371/journal.pone.0161717

7.	Madhusudan M, Karanth K. Local Hunting and the Conservation of Large Mammals in India Local Hunting and the Conservation of Large Mammals in India. 2002;31: 49–54. doi:10.1639/0044-7447(2002)031

8.	Cromsigt, J.P., Kuijper, D.P., Adam, M., Beschta, R.L., Churski, M., Eycott, A., Kerley, G.I., Mysterud, A., Schmidt, K. and West K. Hunting for fear: innovating management of human–wildlife conflicts. J Appl Ecol. 2013;50: 544–549.

9.	Sodhi N, Posa MRC, Lee TM, Bickford D, Koh LP, Brook BW. The States and conservation of Southeast Asian Biodiversity. Biodivers Conserv. 2010;19: 317–328. doi:10.1073/pnas.0703333104

10.	Wegge P, Shrestha R, Flagstad Ø. Snow leopard Panthera uncia predation on livestock and wild prey in a mountain valley in northern Nepal: implications for conservation management . Wildlife Biol. 2012;18: 131–141. doi:10.2981/11-049

11.	Devkota B, Silwal T, Kolejka J. Prey Density and Diet of Snow Leopard (Uncia Uncia) In Shey Phoksundo National Park, Nepal. Appl Ecol Environ Sci. 2013;1: 55–60. doi:10.12691/aees-1-4-4

12.	Oli MK, Taylor IR, Rogers ME. Snow leopard Panthera uncia predation of livestock: An assessment of local perceptions in the Annapurna Conservation Area, Nepal. Biol Conserv. 1994;68: 63–68. doi:10.1016/0006-3207(94)90547-9

13.	Pandey S, Bajracharya SB. Crop Protection and Its Effectiveness against Wildlife: A Case Study of Two Villages of Shivapuri National Park, Nepal. Nepal J Sci Technol. 2016;16: 1–10. doi:10.3126/njst.v16i1.14352

14.	Pant G, Dhakal M, Pradhan NMB, Leverington F, Hockings M. Nature and extent of human-elephant Elephas maximus conflict in central Nepal. Oryx. 2016;50: 724–731. doi:10.1017/S0030605315000381

15.	Neupane B, Budhathoki S, Khatiwoda B. Human-Elephant Conflict and Mitigation Measures in Jhapa District, Nepal. J For Livelihood. 2018;16: 103–112. doi:10.3126/jfl.v16i1.22885

16.	Gulati S, Karanth KK, Anh N, Noack F. Human casualties are the dominant cost of human – wildlife conflict in India. 2020. doi:10.1073/pnas.1921338118/-/DCSupplemental.y

17.	Maskey T, Bauer J, Cosgriff K. Village children, leopards and conservation. Patterns of loss of human live through leopards (Panthera pardus) in Nepal. Kathmandu, Nepal: Department of National Parks and Wildlife Conservation/Sustainable Tourism CRC.; 2001.

18.	Studsrød JE, Wegge P. Park-People Relationships: The Case of Damage Caused by Park Animals Around the Royal Bardia National Park, Nepal°°. Environ Conserv. 1995;22: 133–142. doi:10.1017/S0376892900010183

19.	Adams WM, Hulme D. If community conservation is the answer in Africa, what is the question? Oryx. 2001;35: 193–200. doi:10.1046/j.1365-3008.2001.00183.x

20.	Ministry of Forests and Soil Conservation. Nepal National Biodivesity Strategy and Action Plan 2014-2020. Government of Nepal; 2014.

21.	Silwal T, Shrestha B, Bhatta B, Devkota B. Revenue distribution pattern and park-people conflict in Chitwan National Park, Nepal. Banko Janakari. 2013;23: 35–41. doi:10.3126/banko.v23i1.9465

22.	Niraula R, Gilani H, Pokharel B, Mueen F. Measuring impacts of community forestry program through repeat photography and satellite remote sensing in the Dolakha district of Nepal. J Environ Manage. 2013;126: 20–29. doi:10.1016/j.jenvman.2013.04.006

23.	Adhikari JN, Bhattarai BP, Thapa TB. Human-Wild Mammal Conflict in a Human Dominated Midhill Landscape: a Case Study From Panchase Area in Chitwan Annapurna Landscape, Nepal. J Inst Sci Technol. 2018;23: 30–38. doi:10.3126/jist.v23i1.22158

24.	KC A. Community Forestry Management and its Role in Biodiversity Conservation in Nepal. Global Exposition of Wildlife Management. 2017. doi:http://dx.doi.org/10.5772/65926

25.	Gurung B, Smith JLD, McDougal C, Karki JB, Barlow A. Factors associated with human-killing tigers in Chitwan National Park, Nepal. Biol Conserv. 2008;141: 3069–3078. doi:10.1016/j.biocon.2008.09.013

26.	Acharya KP, Paudel PK, Jnawali SR, Neupane PR, Köhl M. Can forest fragmentation and configuration work as indicators of human–wildlife conflict? Evidences from human death and injury by wildlife attacks in Nepal. Ecol Indic. 2017;80: 74–83. doi:10.1016/j.ecolind.2017.04.037

27.	Bhandari S, Mawhinney BA, Johnson D, Bhusal DR, Youlatos D. Coexistence of Humans and Leopards in Shivapuri Nagarjun National Park , Nepal. Russ J Ecol. 2019;50: 590–592. doi:10.1134/S1067413619060031

28.	Acharya KP. Twenty-four years of community forestry in Nepal. Int For Rev. 2002;4: 149–156. doi:10.1505/ifor.4.2.149.17447

29.	Chauhan NPS. Human casualties and livestock depredation by black and brown bears in the Indian Himalaya, 1989-98. Ursus. 2003;14: 84–87. doi:10.2307/3872960

30.	Kutal D, Kunwar R, Baral K, Sapkota P, Sharma H, Rimal B. Factors that influence the plant use knowledge in the middle mountains of Nepal. PLoS One. 2021; 6–10.

31.	Buchanan Hamilton F. An Account of the Kingdom of Nepal and of the Territories Annexed to the Dominion by the House of Gorkha, Constable, Edinburgh. 1819 (Reprint). Maiijusri, Bibliotheca Himalayica, New Delhi; 1971.

32.	Aryal A, Lamsal RP, Ji W, Raubenheimer D. Are there sufficient prey and protected areas in Nepal to sustain an increasing tiger population? Ethol Ecol Evol. 2016;28: 117–120. doi:10.1080/03949370.2014.1002115

33.	Ayivor J, Nyametso J, Ayivor S. Protected Area Governance and Its Influence on Local Perceptions, Attitudes and Collaboration. Land. 2020;9. doi:10.3390/land9090310

34.	Manfredo J, Michael J, Jerry J, Perry J, J D, Duke EA. Wildlife and Society : The Science of Human Dimensions 1st Edition. Boca Raton: Island Press; 1 edition; 2008.

35.	Rimal B, Sharma R, Kunwar R, Keshtkar H, Stork NE, Rijal S, et al. Effects of land use and land cover change on ecosystem services in the Koshi River Basin, Eastern Nepal. Ecosyst Serv. 2019;38: 100963. doi:10.1016/j.ecoser.2019.100963

36.	Ghimire G, Pearch M, Baral B, Thapa B, Baral R. The first photographic record of the Red Panda Ailurus fulgens (Cuvier, 1825) from Lamjung District outside Annapurna Conservation Area, Nepal. J Threat Taxa. 2019;11: 14576–14581. doi:10.11609/jott.4828.11.12.14576-14581

37.	Kabir M, Ghoddousi A, Awan M. Assessment of human – leopard conflict in Machiara National. Eur J Wildl Res. 2014;60: 291–296. doi:10.1007/s10344-013-0782-z

38.	Constant NL, Bell S, Hill RA. The impacts, characterisation and management of human–leopard conflict in a multi-use land system in South Africa. Biodivers Conserv. 2015;24: 2967–2989. doi:10.1007/s10531-015-0989-2

39.	Gautam A, Thapa B, Pandit B, Dhungana B, Tiewali K, Neupane M, et al. Chitwan-Annapurna Landscape: A rapid assessment. WWF Nepal Program, editor. WWF Nepal, Hariyo Ban Program. 2013.

40.	ICIMOD. Land cover of Nepal 2010 [Data set]. Kathmandu; 2010. Available: https://rds.icimod.org/Home/DataDetail?metadataId=9224

41.	Naha D, Dash SK, Chettri A, Chaudhary P, Sonker G. Landscape predictors of human – leopard conflicts within multi ‑ use areas of the Himalayan region. Sci Rep. 2020; 1–12. doi:10.1038/s41598-020-67980-w

42.	Aryal A, Sathyakumar S, Schwartz CC. Current status of brown bears in the Manasalu Conservation Area, Nepal. Ursus. 2010;21: 109–114. doi:10.2192/09gr029.1

43.	CODEFUND. Profiling of protected and human wildlife conflict associated wild animals in Nepal. DNPWC; 2017.

44.	Bista R, Aryal A. Status of the Asiatic black bear Ursus thibetanus in the southeastern region of the Annapurna Conservation Area, Nepal. Zool Ecol. 2013;23: 83–87. doi:10.1080/21658005.2013.774813

45.	Sharma LK, Charoo SA, Sathyakumar S. Conflict Bear Translocation : Investigating Population Genetics and Fate of Bear Translocation in Dachigam National Park. PLoS One. 2015; 1–17. doi:10.1371/journal.pone.0132005

46.	NTNC. Distribution and Abundance of Himalayan Black Bear and Brown Bearand human-bear conflcit in Manaslu Conservation Area, Gorkha, Nepal. Lalitpur, Nepal: Nepal Trust for Nature Conservation; 2013.

47.	Shah KB, Thapa TB, Budha PB. Status Survey of the Forest Leopard Panthera pardus Linnaeus, 1758 in Nepal. Kathmandu, Nepal; 2004.

48.	Hoare RE. Management implications of new research on problem elephants. Pachyderm. 2015;30: 44–48.

49.	Naha D, Id SS, Rawat GS. Understanding drivers of human-leopard conflicts in the Indian Himalayan region : Spatio-temporal patterns of conflicts and perception of local communities towards conserving large carnivores. PLoS One. 2018; 1–19.

50.	Ministry of Livestock Development. Livestock Statistics of Nepal. Kathmandu, Nepal; 2017.

51.	Annonymous. Man-eating Leopard in Nepal. Cat News. 1989.

52.	DNPWC. Snow Leopard Conservation Action Plan for Nepal 2017-2021. Kathmandu, Nepal: DNPWC and WWF Nepal; 2021.

53.	Thapa TB. Habitat suitability evaluation for Leopard (Panthera pardus) using remote sensing and GIS in and around Chitwan National Park. Saurashtra University. 2011.

54.	DFO. Division Forest Office. Hattisar, Kathmandu: Division Forest Office, Kathmandu; 2013.

55.	ADB. Country Poverty Analysis (Detailed): Nepal. 2017. Available: https://www.adb.org/sites/default/files/linked-documents/cps-nep-2013-2017-pa-detailed.pdf

56.	Storm D, Schauber EM. Deer – Human Conflict and Hunter Access in an Exurban Landscape Deer – human con fl ict and hunter access in an exurban landscape. 2007. doi:10.26077/zs0n-s822

57.	Kunwar RM, Baral K, Paudel P, Acharya RP, Thapa-Magar KB, Cameron M, et al. Land-use and socioeconomic change, medicinal plant selection and biodiversity resilience in far Western Nepal. PLoS One. 2016;11. doi:10.1371/journal.pone.0167812

58.	Miller JRB, Jhala Y V, Jena J, Schmitz OJ. Landscape-scale accessibility of livestock to tigers : implications of spatial grain for modeling predation risk to mitigate human – carnivore conflict. 2015; 1354–1367. doi:10.1002/ece3.1440

59.	Balme G, Hunter L, Slotow R. Feeding habitat selection by hunting leopards Panthera pardus in a woodland savanna : prey catchability versus abundance. Anim Behav. 2007;74: 589–598. doi:10.1016/j.anbehav.2006.12.014

60.	Dhungana R, Savini T, Karki JB, Dhakal M, Lamichhane BR, Bumrungsri S. Living with tigers Panthera tigris: Patterns, correlates, and contexts of human-tiger conflict in Chitwan National Park, Nepal. Oryx. 2018;52: 55–65. doi:10.1017/S0030605316001587

61.	Quammen D. Monster of God: The Man-Eating Predator in the Jungles of History and the Mind. 2004.

62.	Tripathi S, Subedi R, Adhikari H. Forest Cover Change Pattern after the Intervention of Community Forestry Management System in the. Remote Sens. 2020. doi:10.3390/rs12172756

63.	Kshettry A, Vaidyanathan S, Athreya V. Leopard in a tea-cup : A study of leopard habitat-use and human-leopard interactions in north-eastern India. PLoS One. 2017. doi:10.5061/dryad.pc539.Funding

64.	Kissui BM, Kiffner C, König HJ, Montgomery RA. Patterns of livestock depredation and cost ‐ effectiveness of fortified livestock enclosures in northern Tanzania. 2019; 11420–11433. doi:10.1002/ece3.5644

65.	Akhtar N, Chauhan NPS. Status of human wildlife conflict and mitigation strategies in Marwahi Forest Division, Bilaspur Chhattisgarh. Indian Forester. 2008. pp. 1349–1358.

66.	Liu F, McShea WJ, Garshelis DL, Zhu X, Wang D, Shao L. Human-wildlife conflicts influence attitudes but not necessarily behaviors: Factors driving the poaching of bears in China. Biol Conserv. 2011;144: 538–547. doi:10.1016/j.biocon.2010.10.009

67.	Charoo SA, Sharma LK, Sathyakumar S. Asiatic black bear-human interactions around Dachigam National Park , Asiatic black bear – human interactions around Dachigam National Park , Kashmir , India. 2011. doi:10.2307/41304062

68.	Subedi BP., Gauli K, Joshi N, Pandey A, Charmakar S, Poudel A, et al. Forest Carbon Assessment in Chitwan-Annapurna Landscape. Kathmandu, Nepal; 2015.

69.	Baral S, Adhikari A, Khanal R, Malla Y, Kunwar R, Basnyat B, et al. Invasion of alien plant species and their impact on different ecosystems of Panchase Area, Nepal. Banko Janakari. 2017;27: 31–42. doi:10.3126/banko.v27i1.18547

70.	MoFSC. State of Nepals forest. Kathmandu, Nepal: Department of Forests and Soil Conservation; 2015. doi:978-9937-8896-3-6

71.	Reddy CS, Vazeed Pasha S, Satish K V., Saranya KRL, Jha CS, Krishna Murthy YVN. Quantifying nationwide land cover and historical changes in forests of Nepal (1930–2014): implications on forest fragmentation. Biodivers Conserv. 2018;27: 91–107. doi:10.1007/s10531-017-1423-8

72.	Bhattarai BP, Kunwar RM, KC R. Forest certification and FSC standard initiatives in collaborative forest management system in Nepal. Int For Rev. 2019;21: 1–9.

73.	Gill P. Tree-mendous: Community management and outmigration have helped Nepal double its forest area in 25 years. 2019;Sept 13.

74.	FAO. Forest resources of Nepal. Country Report. Rome; 2000.

75.	Adams J, McShane T. The Myth of Wild Africa Conservation Without Illusion: Conservation without illusion. University of California Press, editor. Berkeley; 1994.

76.	Kunwar, RM, Acharya R. Impact Assessment of Invasive Plant Species in Selected Ecosystems of Bhadaure Tamagi VDC , Kaski An Ecosystem-based Adaptation in Mountain Ecosystem in Nepal Impact Assessment of Invasive Plant Species in Selected Ecosystems. Kathmandu: IUCN Nepal.; 2013.

77.	Mishra C. High Altitude Survival: conflcits between pastoralism and wildlife in the Trans-Himalaya. Wagerningin University, The Netherlands. 2001.

Figure 1: Location of study area showing the districts, elevation and points of wildlife attacks. Data for this map was downloaded from ICIMOD [40]. https://doi.org/10.1371/journal.pone.0246390.g001.

Figure 2: Types of HWC resulting in human casualties and injuries.

Figure 3: Temporal distribution of HWCs in study area.

Table 1. Association between socio-demographic, ecological variables and human wildlife conflict. df = degree of freedom.

Table 2. Akaike Information Criterion (AIC) scores of generalized linear models with binomial structure predicting human attacks by wildlife in Parbat, Lamjung, Syangja, Tanahun and Kaski (PLSTK) districts, Nepal. df = degree of freedom, ∆=delta.
