# Supplementary material for: Characterization and management of human-wildlife conflicts in mid-hills outside protected areas of Gandaki province, Nepal
Source: PLoS One. 2021 Nov 19;16(11):e0260307. doi: 10.1371/journal.pone.0260307 (PMC8604285; doi:10.1371/journal.pone.0260307)
Supplement: Attachment — Submitted filename: Response letter5.docx [file pone.0260307.s004.docx]

PONE-D-21-05727
Characterization and management of human-wildlife conflicts in mid-hills outside protected areas of Gandaki province, Nepal
Journal: PLOS ONE

Response to Reviewers comments

We are thankful to Dr. LN Sharma and reviewers for providing us the constructive comments. Please find the details of response to the comments made by reviewers and editors.

1. We suggest you thoroughly copyedit your manuscript for language usage, spelling, and grammar. If you do not know anyone who can help you do this, you may wish to consider employing a professional scientific editing service.

*Please find attached the revised MS language edited by Prof. Dr. Weihong Ji, Massey University New Zealand, and Dr. Craig Morley, TO Institute of Technology, New Zealand. We truly appreciated your cooperation in this regard.*

*Please find the revised MS in both clear and colored font formats for your reference and review. We have added co-corresponding authorship to Mr. Kedar Baral, principal author and PhD student of University of Massey, New Zealand.*

*Details of Corrections:*

*Abstract: Line 24-26, 31-47*

*Introduction: 75-79, 87-89, 95-96*

*Methods: Study area: Line 129-132. Redundant information of Midhills deleted.*

*Line 151-159.*

*Figure 1: Location of study area showing the districts, elevation, land use and points of wildlife attacks. “Republished from [51] under a CC BY license, with permission from ICIMOD, original copyright 2010”.*

*Data collection: 175-177, 180-182,*

*Data analysis: Line 198-202, we shortened the sentences.*

*Table 1. Reorganized column 2 and abbreviation explained.*

*Results: Line 281-281, 284-285, we removed the jargon and sentences were rewritten.*

*Discussion: Line 310-311, Opening sentence reorganized.*

*Line 323-324. Restructured the sentence.*

*Line 332-333, 345-346: Changed the sentences.*

*Line 352-353. Reason explained.*

*Line 365-366, 375: Concluding sentence added.*

*Line 386-388: Sentence restructured.*

*Line 421-426. Sentences rewritten.*

*Line 469-481. Some of the sentences of this section moved to conclusion.*

*Conclusion: Line 492-501. Sentences were restructured and some drawn from discussion section.*

*Reference: Two redundant references were deleted.*

2. Please include the following text in the figure caption: “Republished from [ref] under a CC BY license, with permission from [name of publisher], original copyright [original copyright year]

*Figure 1: Location of study area showing the districts, elevation, land use and points of wildlife attacks. “Republished from [51] under a CC BY license, with permission from ICIMOD, original copyright 2010”.*

3. Please upload a Response to Reviewers letter which should include a point by point response to each of the points made by the Editor and / or Reviewers. (This should be uploaded as a 'Response to Reviewers' file type.) Please follow this link for more information: [http://blogs.PLOS.org/everyone/2011/05/10/how-to-submit-your-revised-manuscript/](http://blogs.plos.org/everyone/2011/05/10/how-to-submit-your-revised-manuscript/)

*We have now uploaded a separate Response letter to editors/reviewers comments. Please find attached.*

Thank you!

Sincerely,

Ripu Kunwar
