# Supplementary material for: Characterization and management of human-wildlife conflicts in mid-hills outside protected areas of Gandaki province, Nepal
Source: PLoS One. 2021 Nov 19;16(11):e0260307. doi: 10.1371/journal.pone.0260307 (PMC8604285; doi:10.1371/journal.pone.0260307)
Supplement: Attachment — Submitted filename: Response to Reviewers Oct 6.13.docx [file pone.0260307.s005.docx]

Oct 6

1. Please upload a Response to Reviewers letter which should include a point by point response to each of the points made by the Editor and / or Reviewers. (This should be uploaded as a 'Response to Reviewers' file type.) Please follow this link for more information: [http://blogs.PLOS.org/everyone/2011/05/10/how-to-submit-your-revised-manuscript/](http://blogs.plos.org/everyone/2011/05/10/how-to-submit-your-revised-manuscript/)

Thank you for suggestion. We tried our best to explain our answer explicitly by point-by-point response method.

2. We note you have resubmitted your manuscript but have not addressed our request regarding the image credit in your Figure 1 caption. At this time, please include the following text in the figure caption completed with the relevant information: “Republished from [ref] under a CC BY license, with permission from [name of publisher], original copyright [original copyright year]”.

Figure 1: Location of study area showing the districts, elevation, land use and points of wildlife attacks. “Republished from [51] under a CC BY license, with permission from ICIMOD, original copyright 2010”.

Oct 13

Additional Editor Comments:

I could see the authors have attempted addressing the comments of both the reviewers. However, it is becoming difficult to track changes in the revised version because of a lot text has been changes after the comments. Moreover, the response to reviewers is not properly provided. Hence, it is imperative that the authors must adhere to the point to point policy. It will be good if the author provide all explanations point to point in the response to reviewers file. One of the reviewer has provided comments in the manuscript which needs to be explained in the response to reviewer file along with the line and page number so the revisions can be tracked.

Thank you Dr. Sharma. We truly appreciate you cooperation and grateful to hear the constructive comments.

Now we collected all the comments (July 15, Aug 28, Oct 6 and Oct 13) and tried our best to address as explicit as possible through point-by-point method. Detail of explanations, texts, along with the line numbers was given.

The comments provided in email and in pdf files both were addressed.
